# The Murine CD137/CD137 Ligand Signalosome: A Signal Platform Generating Signal Complexity

**DOI:** 10.3389/fimmu.2020.553715

**Published:** 2020-12-10

**Authors:** Beom K. Choi, Hyeon-Woo Lee

**Affiliations:** ^1^ Biomedicine Production Branch, Program for Immunotherapy Research, National Cancer Center, Goyang, South Korea; ^2^ Department of Pharmacology, School of Dentistry, Graduate School, Institute of Oral Biology, Kyung Hee University, Seoul, South Korea

**Keywords:** CD137, CD137 ligand, signalosome, bi-directional signal transduction pathways, CD137/CD137L-mediated immune modulation

## Abstract

CD137, a member of the TNFR family, is a costimulatory receptor, and CD137L, a member of the TNF family, is its ligand. Studies using CD137- and CD137L-deficient mice and antibodies against CD137 and CD137L have revealed the diverse and paradoxical effects of these two proteins in various cancers, autoimmunity, infections, and inflammation. Both their cellular diversity and their spatiotemporal expression patterns indicate that they mediate complex immune responses. This intricacy is further enhanced by the bidirectional signal transduction events that occur when these two proteins interact in various types of immune cells. Here, we review the biology of murine CD137/CD137L, particularly, the complexity of their proximal signaling pathways, and speculate on their roles in immune responses.

## Introduction

The mammalian immune system is a highly evolved complex of cells and proteins that defends against intruders (1). It comprises adaptive and innate immune responses carried out by various specific cells (1, 2). Communication between immune cells is critical for initiating, modulating, and maintaining immunity against infections and controlling dysregulated immune responses (3–5). Cytokines produced by immune cells and cell-cell contact are the main means of communication (5, 6). Cell-cell contact *via* adhesion between extracellular proteins and receptor/ligand interactions plays key roles in information flow (3–5). A major example of cell-cell contact in the immune system that we focus on here is the interaction of T lymphocytes with antigen-presenting cells (APCs) (7–9). APCs deliver antigenic information to T lymphocytes. In addition to major histocompatibility complex (MHC)/T-cell receptor (TCR) interaction, costimulatory receptors must associate with their ligands for APCs to achieve antigen-dependent activation of T cells. Upon activation of pattern recognition receptors such as Toll-like receptors (TLRs) by pathogen-associated molecular patterns such as lipopolysaccharide (LPS), APCs mature into functional immune cells that present antigens to T cells. This maturation process involves the expression of molecules such as MHCs, adhesion proteins, ligands for costimulatory receptors, and chemokines (7, 10).

Twenty-nine members of the tumor necrosis factor receptor (TNFR) superfamily (TNFRSF) and 19 members of the TNF ligand superfamily (TNFSF) have been identified. They include OX40/OX40 ligand (L), CD27/CD70, glucocorticoid-induced TNFR-related protein (GITR)/GITRL, and CD137/CD137L pairs, which are all costimulatory receptors and their ligands expressed on T lymphocytes and APCs, respectively (11–13). Ligation of TNFRSF members by the corresponding TNFSF members induces signaling *via* dedicated signal transduction pathways (11, 14). At the same time, TNFSF ligation activates ligand-dependent signal transduction pathways, so-called “reverse signals,” that elicit reverse signal-induced cellular responses (15–17). CD137 (4-1BB, TNFRSF9), a 30-kDa glycoprotein, is an inducible type I TNFRSF transmembrane protein expressed on murine T lymphocytes following antigen recognition by the TCR (12, 18). CD137 ligation evokes survival signals in CD8^+^ T lymphocytes (11, 14). CD137 is expressed in various cells including natural killer (NK) cells, NKT cells, regulatory T cells (Tregs), dendritic cells (DCs), follicular DCs, mast cells, differentiating myeloid-lineage cells, eosinophils, neutrophils, and monocytes (19–23). CD137L, a 34-kDa type II transmembrane glycoprotein, was originally identified as a binding partner to soluble forms of CD137 (sCD137) (24). It is expressed on monocytes, macrophages, B cells, DCs, T cells, differentiating hematopoietic cells, bone marrow cells, and tumor cells (25).

Crosslinking of CD137 with CD137L induces various immune responses involving both adaptive and innate immunity (11, 25–28). In addition, CD137L reverse signals increase the antigen-presenting capacity and inflammation of APCs (29–31). Because binding between CD137 and CD137L on various cells can trigger bidirectional signals, it is difficult to dissect the individual effects of CD137 and CD137L using current experimental tools such as knockout mice and antibodies (Abs) against the two molecules in animal models. **Table 1** summarizes CD137/CD137L-mediated immune responses in mouse models. Although it is accepted that CD137 provides T cells with strong survival signals, it also inhibits CD4^+^ T-cell responses in autoimmune disease models and B-cell responses *in vivo* (44, 48, 64). CD137 knockout results in hyperimmune responses in mice and in hyperproliferation of T cells and myeloid progenitors *in vitro* (27, 40, 41, 65). In contrast, CD137L deficiency lowers CD8^+^ T-cell responses in virus infection models (66, 67) and reduces cytotoxic T lymphocyte activity against vesicular stomatitis virus (27). Reverse signaling *via* CD137L, elicited by treating myeloid cells with recombinant CD137-Fc protein (rCD137-Fc), enhanced myelopoiesis during inflammation (25, 65). In contrast, CD137 deficiency increased the number of myeloid cells *in vitro*, and this effect was abrogated by treatment with rCD137-Fc, implying that CD137L provides negative signals to myeloid cells (27, 65). Even in cell culture, treatment with CD137Ab or rCD137-Fc can influence CD137-mediated cellular responses because certain types of cells express both CD137 and CD137L. Therefore, CD137/CD137L-mediated immune responses appear paradoxical and puzzling. Although plausible explanations have been suggested for the complex effects of mouse CD137/CD137L (mCD137/CD137L), none of the explanations are definitive yet. In this review, we discuss the major factors contributing to the complexity of mCD137/CD137L signals and recent findings that hint at links between the mCD137/CD137L axis and other key immune processes.

**Table 1:** Immune responses generated by CD137/CD137L in animal models.

Mice	Stimulation	Disease model	Outcome	Ref.
**Reduced T cell responses in CD137^−/−^ or CD137L^−/−^ mice**				
C57BL/6	CD137 KO	–	Reduced humoral responses in CD137^−/−^ mice Reduced CTL activity of CD137^−/−^ CD8^+^ T cells against VSV	(27)
C57BL/6	CD137L KO	EAE	Ameliorated EAE symptoms in CD137L^−/−^ mice	(32)
C57BL/6	CD137L KO	Influenza infection	Decreased CD8^+^ T cell accumulation in the lungs and viral clearance and increased mortality in CD137L^−/−^ mice	(33)
C57BL/6	CD137L KO	Chronic MHV-68 model	Impaired cytolytic function and secondary expansion of MHV-68-specific memory CD8^+^ T cells in CD137L^−/−^ mice	(34)
(B6 × 129) F_2_	CD137L KO	LCMV peptide NP_396-404_	Reduced CD8^+^ T cell responses in CD137L^−/−^ mice	(35)
C57BL/6	CD137L KO	LCMV infection	Reduced CD8^+^ T cell expansion in CD137L^−/−^ mice	(36)
C57BL/6	CD137L KO	Influenza infection	Primary CTL response of CD137L^−/−^ mice was normal, but the secondary CTL response was reduced.	(37)
**Enhanced T cell responses in CD137^−/−^ or CD137L^−/−^ mice**				
Balb/c	CD137 KO	CD137^−/−^ MRL/MpJ-Tnfrs(lpr)	Enhanced onset and severity of autoimmune lacrimal gland disease in CD137^−/−^ mice.	(38)
Balb/c	CD137 KO	CD137^−/−^ MRL/MpJ-Tnfrs(lpr)	Enhanced onset and severity of systemic lupus erythematosus in CD137^−/−^ mice.	(39)
C57BL/6	CD137 KO	CD137^−/−^ OT-II	Enhanced clonal expansion of CD137^−/−^ OT-II CD4^+^ T cells	(40)
C57BL/6	CD137 KO	CD137^−/−^ OT-1	Enhanced clonal expansion of CD137^−/−^ OT-I CD8^+^ T cells	(41)
C57BL/6	CD137 KO	Mouse tumor models	Enhanced anti-tumor CD8^+^ T cell responses in CD137^−/−^ mice	(42)
C57BL/6	CD137 KO	–	Enhanced proliferation of CD137^−/−^ T cells *in vitro* Increased myeloid progenitors in CD137^−/−^ mice	(27)
C57BL/6	CD137 KO	MCMV infection	CD137^−/−^ mice have elevated early but reduced persistent MCMV‐specific CD8 responses	(43)
**Suppression of T cell responses by agonistic anti-4-1BB mAb**				
DBA	3H3 clone	Collagen-induced arthritis	Suppression of auto-reactive CD4^+^ T cell responses by CD137 agonist	(44)
Balb/c	2A clone	Fas-deficient MRL/lpr mice	Suppression of lupus-like autoimmune processes by CD137 agonist	(45)
Balb/c	Agonistic anti-CD137	Experimental allergic conjunctivitis	Suppression of experimental allergic conjunctivitis by CD137 agonist	(46)
C57BL/6	3H3 clone	Experimental autoimmune uveoretinitis	Suppression of experimental autoimmune uveoretinitis by CD137 agonist	(47)
C57BL/6	2A clone	Experimental autoimmune encephalomyelitis	Suppression of experimental autoimmune encephalomyelitis by CD137 agonist	(48)
**Enhanced T cell responses by agonistic anti-4-1BB mAb**				
C57BL/6	2A clone	Poorly immunogenic C3, TC-1, and B16-F10	Enhanced T cell responses toward E7 peptide (COPP) by CD137 agonist	(49)
C57BL/6	3E1 & 3H3 clone	GVHD	Increased cytotoxicity against P815	(50)
–	Agonistic	Ag104A & P815	Anti-CD137 mAb eradicates established large tumors in mice *via* CD4 and CD8 T cell responses	(51)
B10.A	3H3 clone	staphylococcal enterotoxin A (SEA)	Enhanced T cell responses toward SEA by CD137 agonist	(52)
(B6 × 129)F_2_	3E1 clone	LCMV peptide NP_396-404_	Agonistic anti-CD137 Ab augmented CD8 T cell responses in CD137L^−/−^ mice similar to those in CD137L^+/+^ mice	(35)
C57BL/6	3E1, 1D8	MCA205, B16, MC38	Anti-CD137 mAb enhances anti-tumor T cell responses, but the mice received lymph node cells from agonistic anti-CD137 mAb-treated animals did not show therapeutic effects.	(53)
DBA	1D8 scFv	1D8 scFv-expressing K1735	Transfected tumor-vaccinated mice rejected established tumor.	(54)
Balb/c RAG2^−/−^	Agonistic 2A	P1A-expressing J558	2A treatment with transfer of P1A-specific CD8 T cells into RAG2^−/−^ mice enhanced rejection of established tumor.	(55)
NOD	Transgenic NOD mice overexpressing membrane-bound agonistic anti-CD137 scFv in pancreatic beta cells	Diabetes	Anti-CD137 scFv on pancreatic beta cell Tg mice developed more severe diabetes than their non-transgenic littermates.	(56)
C57BL/6	CD137-Fc	OT-1 T Tx	CD137 blockade impaired division and IFN-γ secretion of OT-1 CD8 T cells, but not CTL activity.	(57)
–	Agonistic 3H3	Several animal models	CD137 triggering generally enhances CD8 T cell responses, but the therapeutic outcomes are determined by that CD137-mediated alteration in immunity will be beneficial.	(58)
**Suppression of T/B cell responses by agonistic anti-4-1BB mAb**				
Balb/c	1D8 clone	SRBC, huIgG,	Suppression of humoral immunity by CD137 agonist	(59)
Nonhuman primates	Agonistic anti-human CD137 (4B4)	OVA	Suppression of OVA-specific IgG responses by human CD137 agonist	(60)
B6	pMHC II-CD137L Tg	–	Progressive splenomegaly and selective depletion of B cells	(61)
C57LB/6	3H3 clone	*Streptococcus pneumoniae*	Suppression of humoral immunity by CD137 agonist	(62)
C57LB/6	3H3 clone and CD137L KO	*Streptococcus pneumoniae*	Pneumococcal surface protein A-specific antibody responses was suppressed by CD137 agonist and reduced in CD137L^−/−^ mice	(63)

## Major Contributors to the Complexity of CD137/CD137L-Elicited Immune Responses

**Figure 1:**
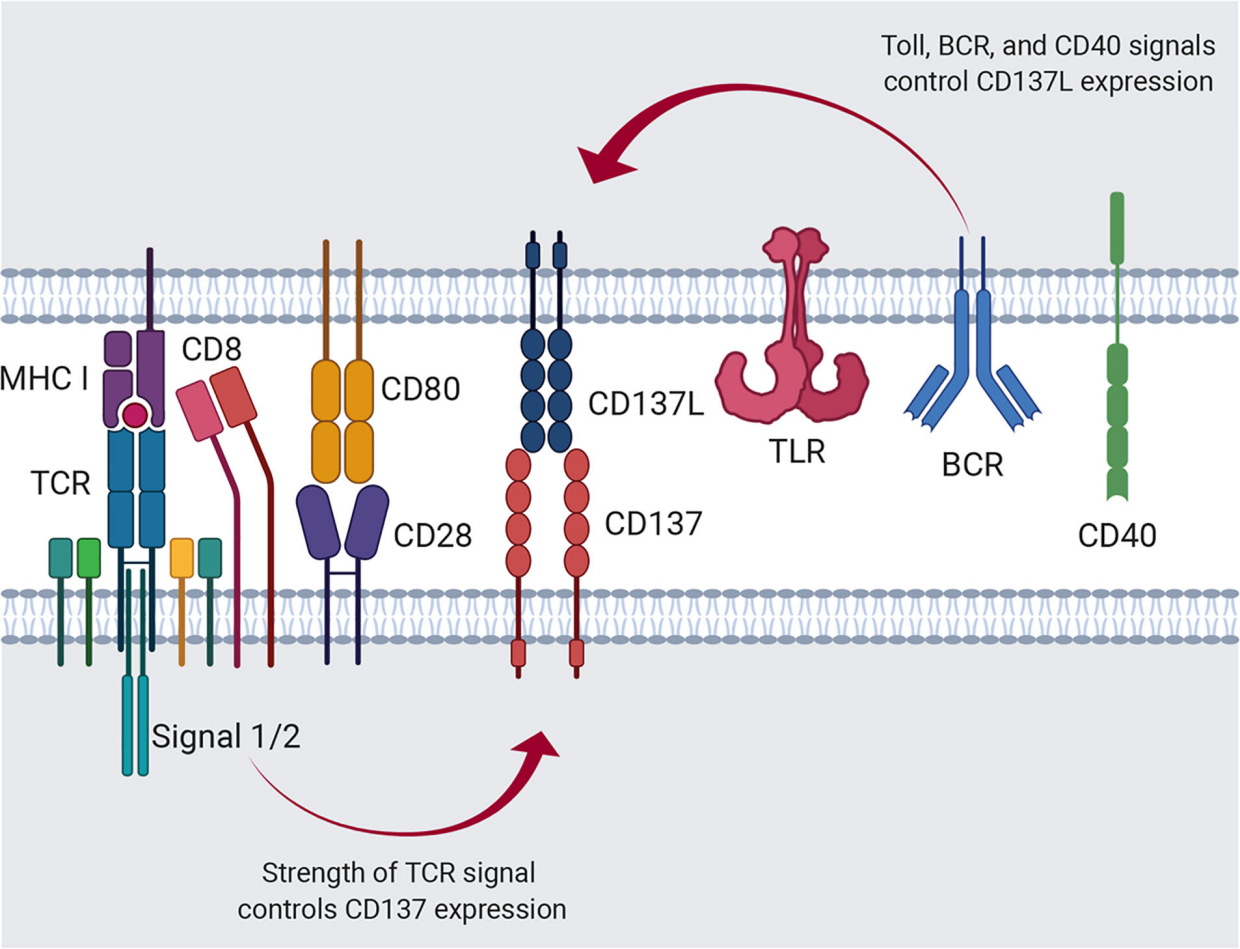
Regulation of CD137 and CD137L expression. Activation of APCs *via* Toll signals, such as TLR4, B-cell receptor crosslinking, and CD40 ligation induces CD137L expression. Alternatively, antigenic signal provided by the MHC I/peptide complex and costimulation through CD28 induces T-cell activation and CD137 expression.

Members of the TNF/TNFR superfamilies, such as CD137L/CD137, evoke dual cellular responses by initiating bidirectional signals (11, 14–17). The interaction between CD137L and CD137 can give rise to a great variety of cellular responses because their expression on both innate and/or adaptive immune cells is induced or enhanced by primary cell activation, such as TCR activation for CD137 and TLR4 activation for CD137L (18, 19, 66–68). For example, resting T cells express little, if any, CD137 (14, 19); however, TCR ligation by antigenic epitopes/MHCs on APCs induces or upregulates CD137 expression on T cells (69, 70). Differences in the affinity and/or avidity of antigenic epitopes for the TCR can influence the level, time of onset, and duration of CD137 expression (**Figure 1**). However, the expression of CD137L on APCs including DCs can be enhanced *via* TLR4 or other receptors in response to stimuli such as LPS (66–68) (**Figure 1**).

The strength and/or type of stimulus may also affect CD137L expression on APCs. Consequently, the CD137/CD137L pair can generate diverse cellular responses depending on the spatiotemporal pattern of their expression and the direction of signaling. Numerous parameters contribute to the complexity of the immune responses elicited by the mCD137/CD137L signalosome. However, in this review, we focus on the bidirectional signal transduction pathways evoked by CD137 and CD137L in the context of T-cell/APC interaction.

### Expression of CD137 and CD137L

mCD137 transcript expression is regulated by multiple elements on the promoter (PI, PII, and PIII). There exist multiple mCD137 transcripts. Types I and II are dominant in the mouse. Type I transcript is preferentially induced in stimulated T cells by TCR activation *via* NF-κB binding on PI. Although type II mRNA is also upregulated in activated T cells *via* AP-1 binding on PI, it is constitutively expressed in various cells *via* AP1 binding on the PI, PII, and PIII elements (71). CD28 costimulation strongly upregulates CD137 expression, presumably *via* binding of both NF-κB and AP-1, as CD28 stimulation sustains TCR signaling pathways such as NF-κB, AP-1, and NFAT (72) (**Figure 1**). The spliced transcript lacking exon 8 encodes sCD137. It is expressed in activated T cells. It has been suggested that sCD137 may antagonize CD137 signal and/or act as an agonist for CD137L reverse signals, and that sCD137 may antagonize CD137 signaling as a decoy receptor (73, 74). However, Tu et al. reported that sCD137 can deliver CD137L reverse signal in CD137L-positive cells such as APCs and T cells (75). They showed that sCD137 protein levels were elevated in adipose tissues of obese subjects, and sCD137 apparently enhanced obesity-induced adipose inflammation by triggering CD137L reverse inflammatory signals in macrophages and activated T cells.

mCD137L expression is induced not only in activated T cells, but also in activated APCs such as LPS-treated macrophages, LPS- or CD40 mAb-treated splenic DCs, and IgM mAb plus CD40 mAb-treated B cells (24, 67) (**Figure 1**). However, the molecular mechanism of its regulation at the promoter level remains uncovered. A recent study by Chang et al. suggested that different APC subsets differentially express costimulatory molecules early during viral infection (76). Classical dendritic cells (cDCs) highly express MHC II, CD80, and CD86, whereas inflammatory monocyte-derived APCs (infAPCs), including inflammatory macrophages and DCs, exhibit the highest expression of TNFSF ligands such as GITRL, OX40L, CD137L, and CD70 (76). Moreover, the authors suggested that GITRL on infAPCs provides signal 4 for activated T cells by providing a post-priming survival checkpoint (76). The results of this study elegantly explained the fundamental roles of inducible costimulatory molecules, including GITR/GITRL, OX40/OX40L, and CD137/CD137L, in the survival of activated T cells.

Another report also indicated that infDCs are a distinct subset of DCs that appear during inflammation and are derived from monocytes that differentiate *in situ* at the site of inflammation and migrate to the lymph nodes to activate T cells, whereas cDCs in lymph nodes generally spend their entire life in secondary lymphoid tissues (77). Given that infAPCs migrate from the site of inflammation while maturing, it is reasonable to expect that infAPCs are mature APCs and cDCs are immature until they start to mature by receiving antigen (Ag) from infAPCs (78). Consequently, inducible costimulatory molecules such as CD137L would be more important for infAPCs than MHC II, CD80, and CD86, which are preferentially expressed on cDCs, because infDCs need to activate T cells and sustain their survival and proliferation.

As mentioned above, CD137 is expressed on activated T cells and CD137L is primarily expressed on myeloid immune cells such as DCs (67). Indeed, when activated CD4^+^ or CD8^+^ T cells were analyzed by flow cytometry, CD137 was readily detected on their surface even 24 h after their activation, whereas CD137L was not (67). CD137L expression was induced on mature DCs following their maturation, but was gradually downregulated as CD137 expression increased (43).

**Figure 2:**
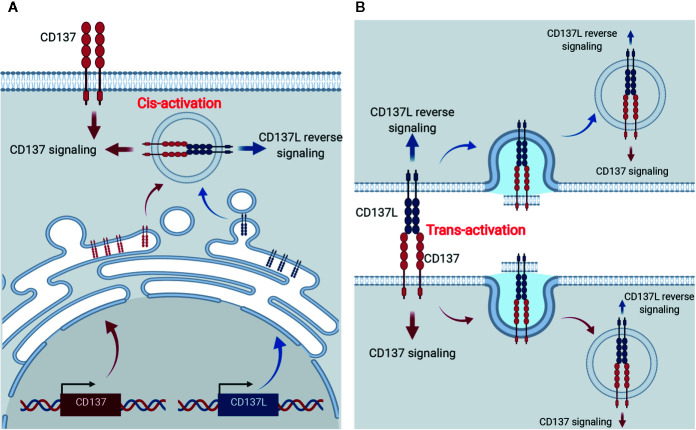
Cis- and trans-activation of CD137 and CD137L. **(A)** Cis-activation of CD137 and CD137L. Newly synthesized CD137 and CD137L interact within activated cells and thus, bidirectional signals are transmitted into the cells. **(B)** Trans-activation of CD137 and CD137L. Following the encounter of activated T cells and APCs, CD137, and CD137L are crosslinked and trogocytosis provoked by activation signals induces internalization of the CD137-CD137L complex and thus, bidirectional signals are transmitted into the cells.

Surprisingly, CD137L expression was enhanced on activated T cells and DCs of CD137-deficient mice (26). It is not clear how T cells and DCs downregulate CD137L during their activation or maturation, but it can be speculated that CD137L becomes bound to intracellular CD137, triggering internalization of the CD137-CD137L complexes, and thus, CD137-mediated signals are continuously transmitted into the cells without a requirement for CD137L located on other cells (**Figure 2A**). Indeed, Martinez-Forero et al. have shown that anti-CD137 mAb bound to CD137 was internalized into an endosomal compartment *in vitro* and *in vivo*, and triggered TRAF2-mediated signaling (79). Since CD137-CD137L complexes are internalized into the cells, theoretically, both CD137- and CD137L-mediated signals can be transmitted into the cells; however, which signal is transmitted *in vivo* remains unclear. Alternatively, CD137:CD137L complexes are exchanged between T:T or T:DC conjugate *via* trogocytosis—the bidirectional transfer of molecules between interacting cells—and thus accumulate in the cells (80) (**Figure 2B**). When activated T cells and mature DCs form conjugates, CD137 and CD137L on each cell interact to form the complex. Since TCR triggering induces trogocytosis (80), molecules on the plasma membrane are exchanged between the conjugated cells and thus, the CD137:CD137L complexes move from donor cells to recipient cells and are internalized and accumulated in the recipient cells *via* receptor-mediated endocytosis. Therefore, it is possible that CD137-mediated signaling in activated T cells is triggered by *trans*-activation of CD137 by CD137L on DCs in early immune responses, but by *cis*-activation of CD137 *via* CD137L in activated T cells in the later phase.

Additionally, if CD137 is more strongly expressed than CD137L in activated T cells or mature DCs, CD137:CD137L complex formation will gradually become saturated, and newly synthesized CD137 begins to be solely located on the plasma membrane. Indeed, CD137 and CD137L mRNA analysis using data from the ImmGen database (www.immgen.org) showed that CD137 is more strongly expressed than CD137L in activated CD8^+^ T cells. This speculation not only provides an explanation for the unsolved question why CD137L is not found on the surface of CD137-expressing T cells or DCs, but also adds complexity to the CD137-CD137L interactions.

### The CD137 Signalosome and Its Signal Transduction Pathways

**Figure 3:**
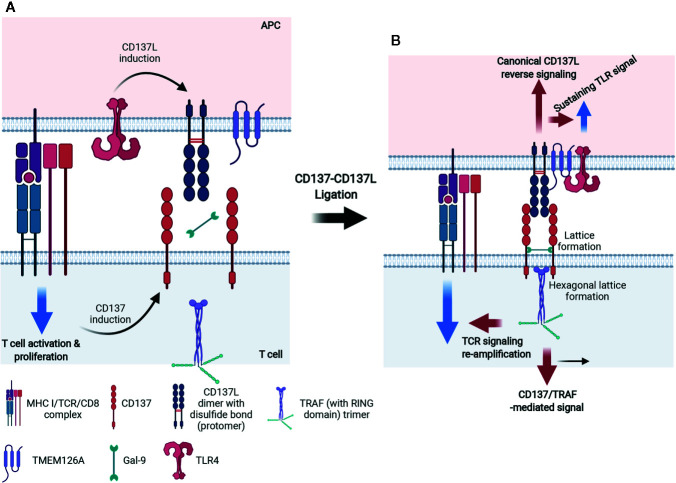
Mechanism of accelerated CD137- and CD137L-mediated signaling in activated T cells and mature APCs. **(A)** For CD137L on APCs, Toll signals, including TLR4, are essentially required and CD137L is expressed and becomes dimerized (protomer) *via* a disulfide bond. Simultaneously, APCs provide antigenic information to T cells by triggering TCR signals. Signal 1 *via* TCR on T cells consecutively induces CD137 expression on T cells. **(B)** After ligation of CD137 and CD137L, CD137 molecules are multimerized and the CD137 multimer is further stabilized by Gal-9 binding, probably through the formation of a lattice structure. The organized CD137 multimer recruits TRAF1/2 and generates TRAF (with a RING domain) trimer. CD137 signaling through the TRAF trimer has two distinct functions: 1) re-amplification of TCR signaling for mitogenic signals and 2) CD137-TRAF-mediated signals for the survival, proliferation, and differentiation of T cells. For CD137 signals, TLR4 and CD137L seem to form a heteromultimer through TMEM126A binding and thus, TLR4 signaling is sustained and canonical CD137L reverse signaling is transmitted.

Although CD137 and CD137L are expressed on various cell types, signaling involving CD137 on T lymphocytes and CD137L on myeloid cells has been studied the most (11, 25). Like other TNFRSF members, CD137 forms a multimer when it binds with CD137L to initiate intracellular signaling. As shown in **Figure 3A**, human (h)CD137L forms noncovalent trimers, whereas mCD137L forms disulfide bond-linked dimers. The hCD137L homotrimer forms three hydrophobic clefts to which the hCD137 homotrimer binds. However, the mCD137 dimeric quaternary structure allows two mCD137 monomers to bind to each mCD137L protomer (81, 82). Recent studies have shown that galectin-9 (Gal-9), a bivalent lectin expressed together with mCD137 on cells, has a critical role in receptor clustering and mCD137-induced proximal signal transduction (81, 83) (**Figure 3B**). mCD137 contains four cysteine-rich domains (CDR1–4) (84) and Gal-9 contains N- and C-terminal carbohydrate-binding domains. An N-glycosylated amino acid in CDR4 is involved in interactions between mCD137 and Gal-9 *via* both the N-and the C-terminal carbohydrate-binding domains (81). This interaction may place the two mCD137 monomers in close vicinity or allow them to cluster, as well as induce a conformational change of the mCD137 cytoplasmic domain that facilitates the initial signaling event, namely binding of TNFR-associated factors (TRAFs) to the cytoplasmic tail of mCD137 (81). Gal-9 also binds to hCD137, which forms a trimeric complex with trimeric hCD137L, and this may induce a conformational change within the cytoplasmic tail of CD137 favoring TRAF binding or clustering (81).

A number of TNFRs are not fully activated by soluble ligands or agonistic Abs, but only by being immobilized or crosslinked. Even ligation of dimeric or trimeric CD137 did not generate effective responses (85). Since multimers of a minimum of nine mCD137 monomers bound to mCD137L nonamer generate functional signals in the absence of Gal-9, it seems that clustering of at least nine CD137 monomers is needed to elicit effective signals (81). It has been suggested that small numbers of ligand-free CD137 molecules are pre-assembled in a two-dimensional hexagonal lattice on the cell surface (86), and that Gal-9 may be involved in the formation of this hexagonal structure (83). Gal-9 may provide a structural platform for CD137 to produce initial signaling events *via* clustering of dimeric or multimeric CD137 (87–90). mCD137L acts together with Gal-9 to aid in the clustering of mCD137 monomers to efficiently initiate mCD137 signaling (81). The Gal-9 lattice may also serve as a docking site for other receptors and signaling molecules (91, 92) (**Figure 3B**). The modulation of TCR responses induced by exogenous Gal-9 depends on the presence of components of the TCR complex such as the tyrosine kinase, Lck (93). There is no evidence that Gal-9 directly interacts with any of the TCR/CD3 components (93). However, it has been suggested that Gal-9 acts on TCR-Lck signals by linking and assembling specific membrane microdomains of T cells, such as lipid rafts (93).

Homo- or heterotrimers of TRAF1 and TRAF2, which are proximal adaptor molecules for the mCD137 receptor signalosome, associate with the cytoplasmic tails of CD137 upon its ligation with CD137L and relay CD137 activation to distal intracellular signaling events, including NF-κB activation, intracellular Ca^2+^ mobilization, and ERK1/2 activation (94–96) (**Figure 3B**). Trimeric TRAFs are made up of combinations of six TRAFs (TRAF1–6); they bind to the cytoplasmic domains of TNFRs and function as scaffold proteins forming complexes with other signaling molecules (97, 98). All TRAFs, except TRAF1, have a RING zinc-finger domain that confers E3 ubiquitin ligase activity, which enables them to regulate the activities of downstream signaling proteins clustered among the TRAFs (99). Homo- or heterotrimers of TRAFs 1, 2, and 3 cluster upon hCD137 activation and stabilize two-dimensional hexagonal lattices of CD137 trimers, forming signalosomes (reviewed in 73). It has been suggested that binding of TRAF2-RING finger dimers as well as cIAP1/2-RING dimers between the TRAF trimers may aid in hexagonal lattice clustering (100–103). In addition, homo- or heterotrimers of TRAFs 1, 2, and 3 recruit various other molecules involved mainly in protein ubiquitination and induce signaling events such as NF-κB, ERK1/2, and p38 MAPK activation [reviewed in (104)].

We have shown that the signaling pathways activated by mCD137 crosslinking are involved in upregulating the expression of anti-apoptotic proteins such as bcl-X_L_, IL-2, and cyclin D2 in murine T cells (14, 105). These proteins are responsible for CD137-mediated increases in cell survival and expansion, which are relevant to the antiviral and antitumor effects of CD137. The early CD137 signaling pathways also help to relocate lipid rafts to the area of contact between T cells and CD137L-expressing cells. Ligation of CD137 leads to the translocation of TCR pathway proteins such as Lck, pTyr, PKC-θ, PLC-γ1, and SLP-76 to lipid rafts (106). Crosslinking of CD137 recruits CD137 and TRAF2 to lipid rafts and re-activates or sustains TCR signaling. We speculate that Gal-9 binding to CD137 creates a platform to induce redistribution of lipid rafts and subsequent recruitment of TCR and/or TCR signaling proteins, as previously described (106). If that is the case, this could be the molecular mechanism underlying reciprocal and continuous regulation of TCR and CD137 signals: the duration of TCR binding by the antigenic epitope/MHC complex on APC would control the expression of CD137, and its ligation by CD137L on APCs would in turn cluster and assemble sufficient Gal-9/CD137 to affect TCR signaling. Thus, it would be important to examine the involvement of Gal-9 in CD137-induced TCR activation.

### CD137L Reverse Signal Transduction Pathways

Crosslinking of mCD137L with rCD137 or anti-CD137L mAb activates reverse signaling. CD137L reverse signal transduction has been studied in human and murine myeloid cells such as monocytes and macrophages (28, 105, 107). In human cells, the CD137L reverse signaling pathways involve Src tyrosine kinase, PI3K, p38MAPK, ERK1/2, and probably, NF-κB. hCD137L may associate with hTNFR1 to initiate these reverse signals (108). In murine macrophages, crosslinking of CD137L increases tyrosine phosphorylation of PP2-sensitive tyrosine kinases such as Src. Subsequently, it activates mTOR/p70S6K, leading to enhanced cell adhesion, survival, and macrophage colony-stimulating factor expression. It also activates Akt *via* Src tyrosine kinase/PI3K, as Akt phosphorylation by CD137L ligation is blocked by Src kinase inhibitor, PP2 or PI3K inhibitors, Wortmannin, and LY294002. CD137L reverse signal-mediated Akt activation is responsible for the upregulation of IL-1β transcript expression 107. Using yeast two-hybrid experiments, we found that CD137L binds to the novel transmembrane protein, TMEM126A, a member of the DUF1370 family of proteins, which consists of several hypothetical eukaryotic proteins with approximately 200 residues. Its biological function is unknown (109). TMEM126A exists as multimers in immune cells, but as monomers in liver cells (109), and associates with the C-terminal extracellular domain of CD137L (109). Binding of TMEM126A to CD137L is a prerequisite for CD137L reverse signaling, in which TMEM126A may play the same role as Gal-9 does in CD137 receptor signaling. In unstimulated myeloid cells, endogenous TMEM126A and CD137L colocalize as widespread spots. Upon crosslinking of CD137 with plate-bound rCD137-Fc, TMEM126A and CD137L colocalized predominantly at the points of contact with the culture plate (109). This suggests that TMEM126A aids in the multimerization of CD137L as well as in the linking of CD137L multimers with other signaling molecules in a CD137L signalosome (**Figure 3B**). CD137L-mediated reverse signals can be as diverse and complex as CD137 receptor signals: a yeast two-hybrid experiment identified proteins and kinases related to ubiquitination, adhesion, and endocytosis as binding candidates (109). In TMEM126A-deficient cells, LPS-induced upregulation of CD54 (ICAM-1), MHC II, CD86, and CD40 expression was diminished, indicating that TMEM126A is involved in TLR4 signaling (110). Flow-cytometric data showed that LPS-induced TMEM126A surface expression increased over time, whereas LPS-induced CD137L expression declined. This implies that TLR4 signaling regulates the cell surface expression of CD137L and TMEM126A. Immunofluorescence data revealed that CD137L/TMEM126A/TLR4 colocalized in spots (109, 110), suggesting that these three proteins may be associated. TMEM126A may also act as a linker between the CD137L signalosome and TLR4 (**Figure 3B**). LPS treatment induces binding of CD137L to TLR4, and their association triggers MyD88-independent TLR4 signals that prolong TNF production (111, 112). It will be important to examine whether TMEM126A interacts directly with TLR4 as it does with CD137L. This would support the hypothesis that TMEM26A bridges the CD137L signalosome with TLR4. Activation of TLR4 by LPS increases the expression of CD137L and TMEM126A on APCs, producing reverse signals that strengthen innate immune cell function. At the same time, CD137L reinforces TLR4-induced responses, presumably by binding to TMEM126A. In other words, mutual interactions take place between CD137L and TLR4 in APCs, as they do between the CD137 signalosome and TCR in T cells.

### Biased Receptor Signaling

**Figure 4:**
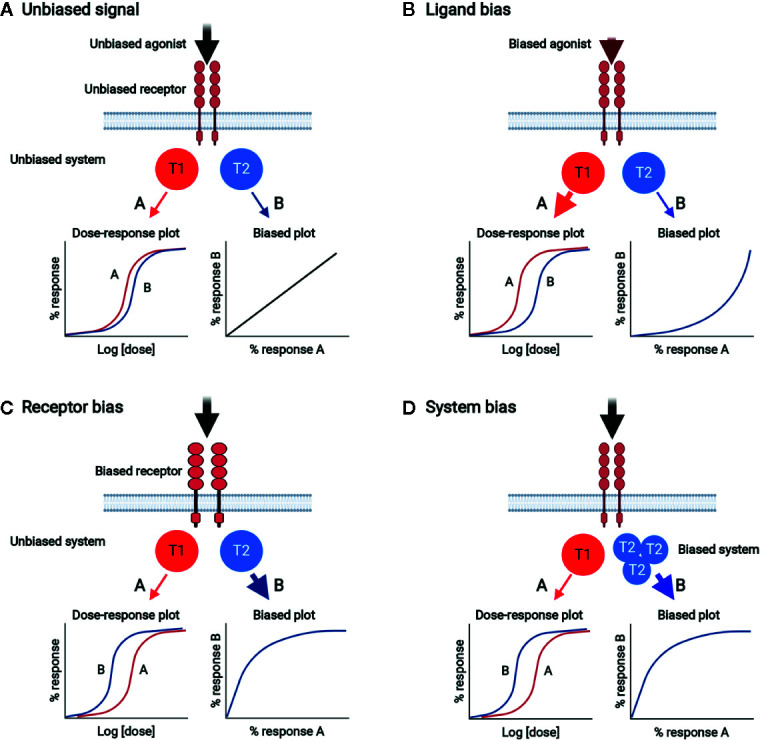
Biased signal generated by ligand bias, receptor bias, or system bias. **(A)** Unbiased signal. Unbiased agonist binds to unbiased receptor, equally activating signal pathways A and B through unbiased transducers T1 and T2, respectively. The dose-response plot shows the equivalent potencies for pathways A and B. Bias plot displays unbiased signal. **(B)** Ligand bias. Biased agonist binds and selects a certain unbiased receptor conformation, which relays signal dominantly through T1 rather than T2. The dose-response plot shows that the potency of response *via* pathway A is stronger than that *via* pathway B. The bias plot indicates that the signal is biased toward A. **(C)** Receptor bias. Unbiased agonist binds to biased receptor, which drives signal to pathway A rather than pathway B. The dose-response plot shows that the potency of response *via* pathway B is stronger than that *via* pathway A. The bias plot indicates that the signal is biased toward B. **(D)** System bias. Unbiased agonist binds to unbiased receptor, interacting with and activating more T2 than T1. Signal *via* pathway B is preferred over that *via* pathway A. The dose-response plot shows that the potency of response *via* pathway B is stronger than that *via* pathway A. The bias plot indicates that the signal is biased toward B.

Plenty of experimental evidences support the notion that multiple receptor conformations exist, and ligands can select and stabilize the different receptor active states for signal transmission (113–117). In this way, one signal can be selectively produced over others (biased signaling). While signal bias has been extensively studied for G protein-coupled receptors, theoretical and experimental data suggest that it is a general behavior of ligands and receptors (114, 118, 119). Signal bias can be generated by any of the three components of the ligand/receptor/transducer complex, generating ligand bias, receptor bias, and system bias, respectively (**Figure 4**). Biased signaling through stabilization of select, multiple conformations of the receptor with a wide range of signaling effector molecules can be used in normal physiology to achieve fine control in naturally healthy body systems (116). Ligand bias refers to ligand-induced signal bias through the selection (or induction) of a unique receptor conformation coupled with preferentially adopted transducers. Receptor bias can be produced by altering the receptor to change its ability to bind to specific ligands and/or activate transducers. Receptor mutation and alternative splicing can generate biased signal (117). Receptor homodimerization can produce functional complexity associated with binding and activation cooperativity originating from allosteric interactions between the protomers making up an oligomer and intrinsic ligand efficacy (116). Moreover, in the receptor homodimer context, ligand bias changes with ligand concentration (116, 119). System bias can be established by changing the context of transducers/effectors, such as their expression levels. Since different cell and tissue types have different signaling systems, certain ligands acting on the same receptor can have different signaling properties depending on the cell type, and each receptor can produce unique responses with same ligand because of system bias.

### Biased Signaling in CD137/CD137L Bidirectional Pathways

**Figure 5:**
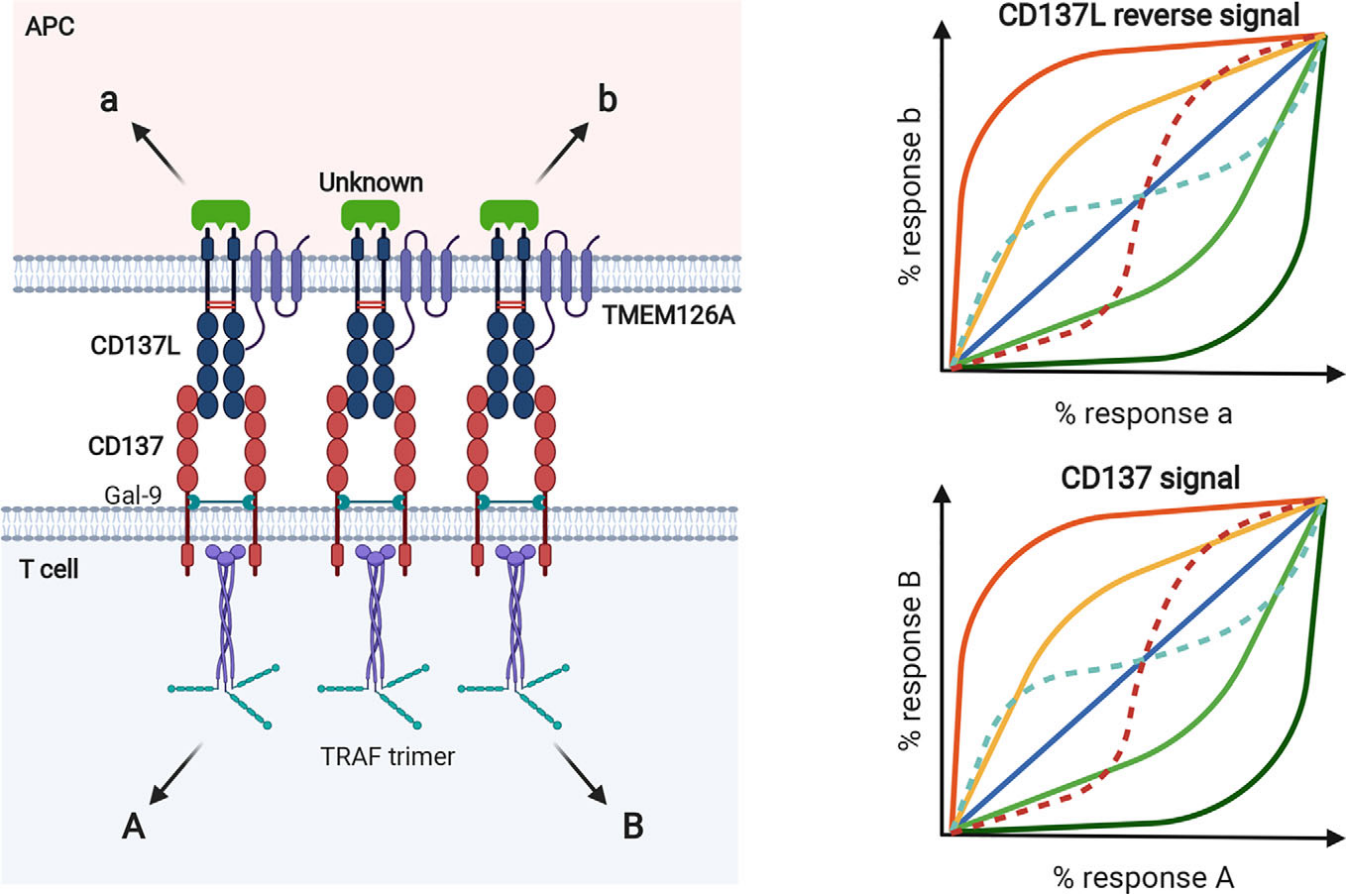
Biased signal in murine CD137/CD137L bidirectional signaling pathways. Expression levels of CD137 and CD137L and their extent of homodimer clustering can establish receptor and ligand bias in T cells and APCs, respectively. In terms of bidirectional signaling, receptors can be ligands for reverse signal and *vice versa*. Species and levels of transducers, such as TRAF trimer, can generate system bias. Because levels of CD137 and CD137L expression are transcriptionally and posttranslationally regulated by Ag exposure, various cellular responses can be generated by bidirectionally biased signals as shown in the bias plot. In the receptor homodimer context, ligand binding can generate receptor bias *via* allosteric interactions between protomers, through which ligand binding affinity and/or activation of downstream transducers can be altered. Ligand bias can also change with ligand concentration in the receptor homodimer (dotted lines in the bias plot).

In clustered dimeric CD137/CD137L signalosome complex, the expression dynamics of CD137 and CD137L may be important to establish biased signals in a cell. For example, antigen species, duration of antigenic challenge, and/or antigen levels determine their expression levels. The levels, degrees, and species of clustering homodimer of CD137 or CD137L and proximal downstream signaling molecules, such as TRAFs for CD137 and their unknown counterparts for CD137L, may generate receptor bias and ligand bias, as well as system bias. That is, the same CD137L (or CD137) can generate different and unique CD137 signals (or CD137L reverse signals) in a cell depending on antigenic differences. As mentioned above, receptor homodimerization confers receptor bias *via* allostery-mediated cooperativity for receptor binding and/or activation of downstream transducers (116). This notion is supported by a recent finding that the mCD137L dimeric structure undergoes allosteric changes upon mCD137 binding (82). Receptor homodimerization may also change the pattern of ligand bias with ligand concentration. Along with the intrinsic complexity of the homodimeric CD137/CD137L bidirectional signalosome, steric and topological changes in conformation induced by CD137/CD137L binding in the clustered, multimeric state may lead to the diversity and complexity of receptor/ligand-induced cellular responses (**Figure 5**). In addition, cellular diversity in expression of CD137/CD137L and transducers and their interactions with other key signaling receptors such as TCR and TLR4 further complicate CD137/CD137L bidirectional signals and related responses. These complexities established by signal bias in CD137*/*CD137L bidirectional signaling pathways may be optimal for finetuning of the immune response. We assume that CD137/CD137L signalosome-mediated *in vivo* cellular responses in the immune system may not be completely understood because the experimental tools used to elucidate CD137/CD137L functions, such as knockout mice, Abs, and recombinant proteins, can interfere with the complex organization of CD137/CD137L bidirectional signalosomes.

## Complexity of CD137/CD137L Signal-Mediated Responses *In Vivo*

The complexity of CD137/CD137L signaling pathways in T cells and APCs *in vivo* is even more complex. CD137^−/−^ B6 mice display a reduced humoral response to KLH and reduced cytotoxic T-lymphocyte activity against vesicular stomatitis virus (27). In line herewith, CD137L^−/−^ mice show defects in inducing CD8^+^ T-cell responses against viral infections, such as influenza (33), MHV-68 (34), and LCMV (35), and further study revealed that secondary rather than primary CD8^+^ T-cell responses against influenza infection are impaired in these mice (120). Because CD137/CD137L signals augment T-cell responses by preventing activation-induced cell death (AICD) (14), stimulating cell-cycle progression (105) and Th1 responses (121), and accelerating metabolism (122), these data fit the expectation that deficiency of CD137 or CD137L *in vivo* reduces T-cell immunity.

Although CD137^−/−^ and CD137^−/−^ mice exhibit reduced CD8^+^ T-cell responses in viral infection models (33–35), their T-cell responses in spontaneous autoimmune disease and tumor models are somehow enhanced (42). CD137 deficiency in MRL/lpr mice accelerated the development of lacrimal gland and skin lesions, enhanced lymphadenopathy, and led to early death, along with increased CD4^+^ T cells and B-cell activity (38, 39). Tumor growth rates were reduced in CD137^−/−^ mice compared to WT B6 mice in an NK- and CD8^+^ T cell-dependent manner (42). Moreover, adoptively transferred CD137^−/−^ OVA-specific OT-I and OT-II cells hyperproliferated in WT B6 mice (26, 41), and CD137^−/−^ mice had increased numbers of primary CD8^+^ T cells and fewer memory CD8^+^ T cells during chronic/latent infection with mouse CMV (79). These data indicate that the CD137/CD137L axis is capable of both stimulatory and inhibitory cosignaling activities *in vivo*; however, the mechanisms underlying the enhanced T-cell responses in CD137^−/−^ mice are not completely understood. In humans, CD137 deficiency is highly correlated with autoimmunity, autoimmune lymphoproliferation, common variable immune deficiency, and malignancies (123).

Agonistic anti-CD137 mAb also has dual stimulatory and inhibitory cosignaling activities *in vivo*. Agonistic anti-CD137 mAb typically enhances CD8^+^ T-cell responses against immunizing peptides such as HPV E7 (49) and LCMV NP_396-404_ (35), staphylococcal enterotoxin A (51), and several mouse tumor cells including Ag104A, P815, MC38, and B16-F10 melanoma (51, 53). The oxygen-deprived tumor microenvironment (TME) upregulates CD137 expression *via* the transcription factor HIF-α in CD4^+^ and CD8^+^ tumor-infiltrating T lymphocytes. Hypoxia-induced CD137 expression in tumor-infiltrating T lymphocytes is thought to explain why intratumoral injection of agonistic anti-CD137 mAb can achieve immunotherapeutic antitumor effects with minimum systemic side effects (124). Since triggering the CD137 signal on CD8^+^ T cells has therapeutic potential in cancer, agonistic anti-CD137 mAbs are under investigation as immunotherapeutic agents in cancer (125, 126). CD137 has been recognized as one of the critical immune checkpoint molecules for tumor Tregs. However, its role in Tregs remains controversial: some studies have suggested that CD137 mAb can block Treg activity (127, 128), whereas one study reported that it enhanced Treg proliferation (129). Recent studies have shown that depletion of Tregs in the TME by depleting anti-CD137 mAb decreased tumor growth, indicating that Tregs in the TME express CD137 to suppress antitumor hyperimmune responses (72, 130). Agonistic anti-CD137 mAb also reduces the incidence and severity of autoimmune diseases including experimental autoimmune encephalitis, collagen-induced arthritis, systemic lupus erythematosus, and experimental autoimmune uveoretinitis (44, 45, 47, 48).

**Figure 6:**
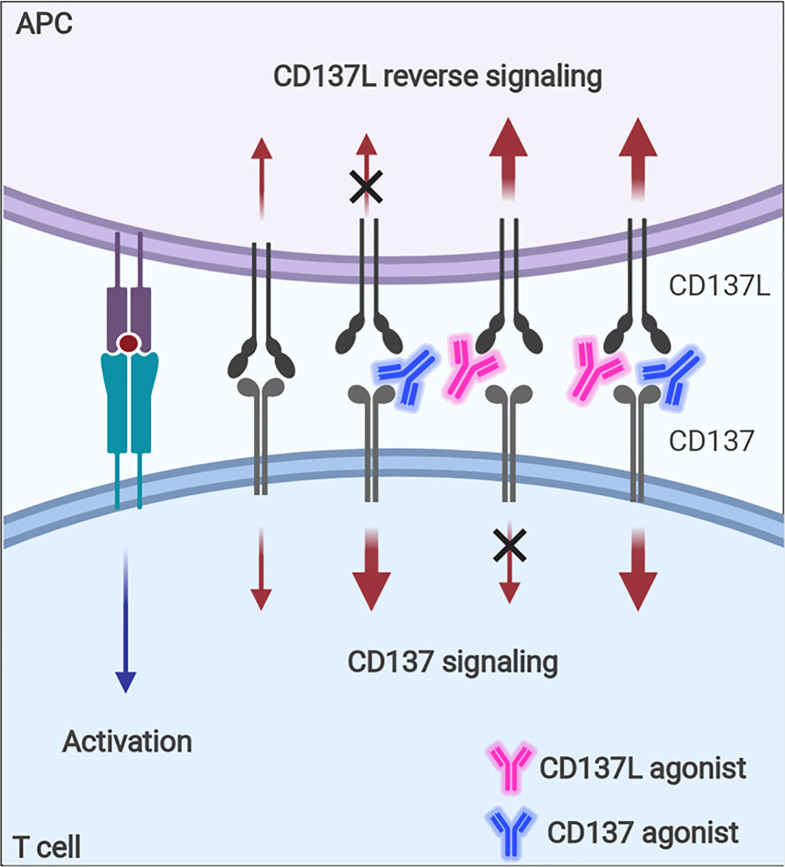
Immune modulation with CD137 or CD137L agonist. Signaling *via* CD137 or CD137L into activated T cells and APCs can be modulated with agonistic mAb. Since the ligation of CD137 and CD137 transmits bidirectional signals, CD137 agonist will enhance CD137 signal, but block CD137L-mediated signal, whereas CD137L agonist will have the opposite effects.

Although CD137 signaling itself seems to be multifaceted in terms of immune response modulation, the contrasting immune responses found in tumor-challenged CD137^−/−^ mice—i.e., enhanced NK- and CD8^+^ T-cell responses against tumors—need to be fully understood because agonistic anti-CD137 mAbs are being evaluated as antitumor therapeutics based on their positive effects on CD8^+^ T cells (125, 126). It seems that the enhanced T-cell responses in CD137^−/−^ mice are due to intrinsic effects accumulated during the development of the immune system in the absence of CD137 signals and thus, cannot be simply mimicked when CD137 signals are blocked with antagonistic anti-CD137 mAb *in vivo*. In addition, since the ligation of CD137-CD137L induces bidirectional signals, agonistic mAb of CD137 or CD137L will enhance the signal *via* its target, but block the signal through its counterpart (**Figure 6**), which also increases the complexity of CD137-CD137L signaling.

## Concluding Remarks

**Figure 7:**
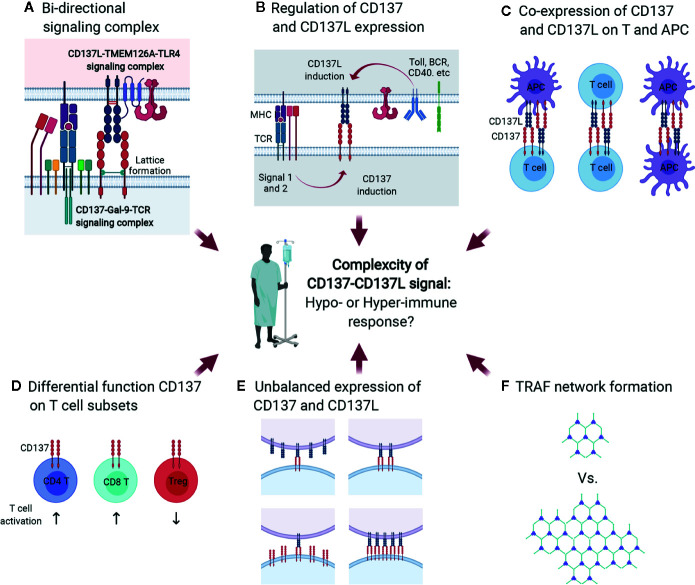
Multiple factors contribute to complexity of CD137-CD137L signal. **(A)** Bidirectional signaling complex. During direct interaction of activated T cells and APCs, CD137-Gal-9 complex and CD137L-TMEM126A-TLR4 complex simultaneously transmit signals. **(B)** Multiple factors, including Ag affinity, costimulatory signals, and APC maturation triggers, are involved in CD137 and CD137L expression. **(C)** Co-expression of CD137 and CD137L. CD137 and CD137L are well known to be expressed on activated T cells and mature APCs, respectively. However, a recent study demonstrated that CD137L is also expressed in activated T cells. Therefore, CD137 and CD137L can interact not only in the T cell-APC conjugate, but also in T cell-T cell or APC-APC conjugates. **(D)** Differential functions of CD137 in T-cell subsets. CD137 triggering induces proliferation and anti-apoptosis in CD4 and CD8 T cells and Tregs. CD137 triggering enhances CD4 and CD8 T cell-mediated immunity and induces Treg-mediated immune suppression because CD137 signal expands activated Tregs that can suppress the overall immune response. **(E)** Unbalanced expression of CD137 and CD137L. **(F)** Limited ligation of CD137 and CD137L leads to inefficient formation of the TRAF network that is required to re-amplify TCR signal and CD137-mediated signal.

Since CD137L is an endogenous ligand that binds to CD137, it is conceivable that the CD137/Gal-9/TCR signaling complex may interact with the CD137L/TMEM126A/TLR4 signaling complex at immunological synapses (**Figure 7A**), which are nanoscale gaps between T lymphocytes and APCs. In signalosome complexes at immunological synapses, these six proteins may influence one another by modulating each other’s expression level, provoking CD137/CD137L bidirectional signaling, and modifying TCR/TLR4 signaling during APC maturation and subsequent antigen presentation to T cells at peripheral infection sites or in lymphoid organs. Encounters with pathogen-associated molecular patterns (and/or damage-associated molecular patterns) activate pattern recognition receptors on APCs such as TLR4 at peripheral sites, and this usually induces pro-inflammatory responses as well as APC maturation involving increases in the expression of MHC proteins, adhesion molecules, and costimulatory molecules. During APC maturation, CD137L expression is upregulated together with TMEM126A expression (**Figure 7B**), and CD137L may adopt a specific conformation by forming a complex with TMEM126A and TLR4. This conformation may be needed for prolonged activation of TLR4, as well as for initiating CD137L reverse signals upon engagement with CD137 on T cells at a later time when APCs encounter T cells in lymphoid organs. CD137L reverse signals induce further maturation of APCs, which enhances antigen presentation to T cells. When just-matured APCs meet T cells in lymphoid organs, the MHC/epitope on the APCs binds to the TCR on the T cells, and TCR signals are produced with the help of costimulatory molecules such as CD28/B7. TCR activation induces or enhances CD137 expression (**Figure 7B**). CD137 then interacts with Gal-9 and adopts a state that enables it to bind to a suitable form of CD137L and initiate CD137 signals. In the complex, crosslinking of CD137/Gal-9 re-recruits TCR signaling molecules and maintains TCR signaling, which promotes the differentiation of naïve T cells to effector and/or memory T cells.

The hyperproliferation of CD137-deficient T cells may be due to CD137/CD137L-induced suppression of T-cell proliferation. CD137L reverse signals suppress T-cell activation (29, 131) (**Figure 7C**). Alternatively, highly expressed CD137 generated by TCR activation with high-affinity Ag may form complexes with TCR/Gal-9. The conformation of TCR/Gal-9/CD137 adopted upon contact with CD137L may tune TCR signals to block their hyperproliferation, but promote their survival and differentiation. Either way, CD137/CD137L may be able to induce optimal T-cell responses during antigen contact. The *in vivo* situation may be even more complex. CD137 and CD137L bidirectional signals may affect the expression and/or signaling pathways of other costimulatory proteins and/or cytokines. Moreover, TLR4 and TCR signals modified by CD137/CD137L signals may influence other molecules at immunological synapses. Signal bias due to the clustering of dimerized CD137/CD137L, CD137/CD137L expression levels and duration, the degree of binding with cis proteins such as Gal-9, TRAFs, and TMEM126A, and interaction with other key proteins such as TCR and TLR4, gives rise to the complexity of CD137/CD137L bidirectional signalosome-mediated cellular responses (**Figures 3** and **7C**).

In general, CD137 signal positively regulates T-cell responses. However, in the case of Tregs, CD137 signal not only increases number of Tregs by enhancing their proliferation, but also temporally neutralizes the suppressive function of activated Tregs, but not naïve Tregs (47). Since CD137 triggering increases the numbers of Tregs and transiently neutralizes their suppressive activity, overall T-cell responses will be strongly inhibited by the suppressive activity of the increased Tregs when CD137L is discontinued (**Figure 7D**). Therefore, although CD137 signal exerts similar effects such as proliferation enhancement and anti-apoptosis on T-cell subsets, the outcomes of CD137 triggering can differ depending on the function of the T-cell subsets, which increases the complexity of CD137/CD137L signaling.

Another factor contributing to the complexity of CD137/CD137L signaling is unsynchronized CD137 and CD137L expression. Multiple factors, including TCR strength, costimulation, and Toll ligand, are involved in CD137 and CD137 expression on T cells and APCs. If the expression of these molecules is not spatiotemporally synchronized, unbalanced CD137 and CD137L expression may lead to limited ligation of CD137 and CD137L, eventually resulting in inefficient TRAF network formation and delayed T-cell activation (**Figures 7E, F**).

Immunity against danger signals may be controlled by these web-like interactions. It is difficult to systemically dissect this complexity. However, it is clear the complexity of the molecular interactions gives rise to the immunity required to protect us from the many menaces, and that derangement of the interactions may lead to hypo- or hyperimmune status and to immunopathogenic conditions such as infective inflammation, autoimmune diseases, and possibly, tumors. This may be why contradictory responses have been reported in disease models when anti-CD137 mAb or rCD137L was administered. For example, viral infection or tumors that can escape immune surveillance may create conditions that inhibit the association of CD137 with CD137L and thus suppress T-cell activation. Ligation of T-cell CD137 with agonistic anti-CD137 Ab or rCD137L may reverse the T-cell inhibition induced by viral infection or tumors. In contrast, hyperimmunity in autoimmune diseases may be caused by strong association of CD137 with CD137L. CD137 Abs may restrain this and dampen the CD137/CD137-mediated signals in T cells and APCs.

Sophisticated *in vivo* mouse experiments using natural antigenic challenge and monitoring changes in mCD137/CD137L expression, localization, and steric structure should provide some answers to the following questions: 1) How are signalosome complexes formed? 2) What is the driving force behind their formation? 3) How do they provide the array of intracellular signals needed for dealing optimally with host immunological cues?

## Author Contributions

H-WL and BC equally contributed to plan a scheme of this review. H-WL reviewed on bi-directional signaling and proposed potential roles of biased signal in CD137/CD137L complexity. BC reviewed and described about *in vivo* immune responses mediated by CD137/CD137L and their expression regulation. All authors contributed to the article and approved the submitted version.

## Funding

This work was supported by a National Research Foundation of Korea (NRF) grant funded by the Korea government (MSIT) (2020R1F1A104819111) to H-WL and a grant of the National Cancer Center of Korea (NCC-191050) to BC.

## Conflict of Interest

The authors declare that the research was conducted in the absence of any commercial or financial relationships that could be construed as a potential conflict of interest.
